# Information and Perception of Meaningful Patterns

**DOI:** 10.1371/journal.pone.0069154

**Published:** 2013-07-19

**Authors:** Maria M. Del Viva, Giovanni Punzi, Daniele Benedetti

**Affiliations:** 1 NEUROFARBA Dipartimento di Neuroscienze, Psicologia, Area del Farmaco e Salute del Bambino Sezione di Psicologia, Università di Firenze, Firenze, Italy; 2 IMB University of Chicago, Chicago, Illinois, United States of America; 3 Dipartimento di Fisica “E. Fermi” Università di Pisa, Pisa, Italy; 4 Fermi National Accelerator Laboratory, Batavia, Illinois, United States of America; SUNY Downstate MC, United States of America

## Abstract

The visual system needs to extract the most important elements of the external world from a large flux of information in a short time for survival purposes. It is widely believed that in performing this task, it operates a strong data reduction at an early stage, by creating a compact summary of relevant information that can be handled by further levels of processing. In this work we formulate a model of early vision based on a pattern-filtering architecture, partly inspired by high-speed digital data reduction in experimental high-energy physics (HEP). This allows a much stronger data reduction than models based just on redundancy reduction. We show that optimizing this model for best information preservation under tight constraints on computational resources yields surprisingly specific a-priori predictions for the shape of biologically plausible features, and for experimental observations on fast extraction of salient visual features by human observers. Interestingly, applying the same optimized model to HEP data acquisition systems based on pattern-filtering architectures leads to specific a-priori predictions for the relevant data patterns that these devices extract from their inputs. These results suggest that the limitedness of computing resources can play an important role in shaping the nature of perception, by determining what is perceived as “meaningful features” in the input data.

## Introduction

The visual system needs to extract the most important features in the environment from a large flux of information in a short time for survival purposes. In fact, rapid and reliable detection of visual stimuli is essential for triggering autonomic responses to emotive stimuli, for initiating adaptive behaviours and for orienting towards potentially interesting, or dangerous, stimuli [1]. The speed of visual processing can be as low as 100 ms for animals and face processing [2], reduced to 30 ms when images present affective contents [3].

The amount of information that needs to be processed in that limited amount of time is quite significant. Based on the number of neurons and their capacity of transmission, Echeverry [4] estimated the capacity of transmission of photoreceptors (9.2·10^7^ rods and 4.6·10^6^ cones) around 20 Gb/s for each eye, reduced to 4 Gb/s at the level of optic nerve fibers (approximately 10^6^ for each eye), with a neural ratio of nearly 124∶1. Comparing these data with the limits on the brain’s capacity to process visual information imposed by intrinsic costs of neuronal activity involved in cortical computation [5] [6] (largely dependent on the rate at which neurons produce spikes [7]) highlights the existence of information bottlenecks [8]. It is therefore widely believed that the visual system operates by performing a strong data reduction at an early stage [9], [10], creating a compact summary of relevant information that can be handled by further levels of processing. Independently motivated models describe the initial processing of visual information as the extraction of a simplified “sketch” based on a limited number of “salient features” [11], [12], that therefore contains a much reduced amount of information.

Past studies have shown that some known properties of early vision can actually be understood in terms of efficient coding of information by reducing redundancy [10], [8], [13]. These models however take an approach based on preserving the majority of the available information, and do not lead to large reduction factors, with extraction of few salient features. Conversely, existing successful models of how the visual system extracts salient features are based more on the a-posteriori knowledge of specific physiological details, than on considerations of information compression efficiency [14]–[16].

In the present work, we discuss a model of early visual processing, aimed explicitly at reducing information significantly through a selection of salient features. Our model is based on some very general assumptions. First, we impose a tight upper bound on the total amount of data that can be produced as output. Second, we assume that there is only a fixed number of pre-determined *patterns (visual features)* that the system can recognize in its input. Third, that the reduction of data flow is achieved by filtering only those pieces of input data matching this reference set of patterns, disregarding any other information. Our model is purely functional: we do not concern ourselves with the details of how this computation is implemented.

This allows the model to be applied to a wide range of problems. The need for extracting a small amount of “relevant” information from a large input flux of data is certainly not unique to vision [17], although vision may be one of the fields where the requirements are particularly severe. An interesting example in a very different field is data acquisition in experimental particle physics (HEP). Experiments in this field often require the collection of data output from large particle detectors, exceeding O(Tbyte/s) for extended periods of time (years). Cost and practical considerations limit the flux of data that can be stored for later analysis to factors of thousands less data, and as a consequence, the typical operating mode of these experiments is based on a real-time selection of a very small fraction of data that are deemed “interesting” and saved for later analysis [18].

In particular, the common HEP problem of recognizing the trajectories of particles crossing the detector, starting from a set of measured points, is quite similar to the problem faced by the visual system in extracting meaningful elements of the external word, starting from a luminance map on the retina. It is interesting to note that in HEP experiments, a very effective solution to this problem [18], [19] has been achieved through specialized VLSI electronic devices (Associative Memories) [20], which allow for massively parallel pattern-matching at high speed, implementing a pattern-filtering functionality precisely of the type we have defined above in general terms as our reference model.

The functionality of our abstract pattern-filtering model is completely defined by its reference set of *patterns*. This also means that our discussion does not need to be concerned with the specific computation used in their recognition, nor with its localization within any specific anatomical structure. However, the number of possible a-priori choices for a set of visual patterns is very large, and in order to make any progress in developing and testing our model we must determine it precisely. Possible approaches are to appeal to known properties of neuron receptive fields, or to considerations of performance in the reconstruction of visual scenes; they have often been used in developing similar models in literature. In experimental HEP applications, the appropriate patterns are determined from a detailed knowledge of the detectors and the laws governing the motion of particles.

We have chosen a different approach, focusing only on the requirement to be information-efficient. We assume that the system is optimal from the point of view of delivering the maximum amount of information to the following processing stages. This idea has already had some successful applications in redundancy reduction [8]; we apply it here to the selection of features. We adopt the principle of maximum entropy as a measure of optimization: we ask what is *choice of the pattern set producing the largest amount of entropy allowed by the given limitations of the system*. We will see that this simple requirement, together with the imposed strict limitations to the computing resources of the system, allows to completely determine the choice of the pattern set from the knowledge of the statistical properties of the input data. This allows us to make detailed predictions with very few tunable parameters, and to compare them with the real behavior of both human viewers, and artificial systems for data acquisition in HEP experiments. The latter can provide useful insight, as their structure is fully known in all details.

## Materials and Methods

### Ethics Statement

For research involving human participants the data were analyzed anonymously.

All subjects were aware of the purposes of the study and gave written informed consent.

The research was approved by the local ethics committee of the Department of Psychology of University of Florence.

### Model

**Figure 1:**
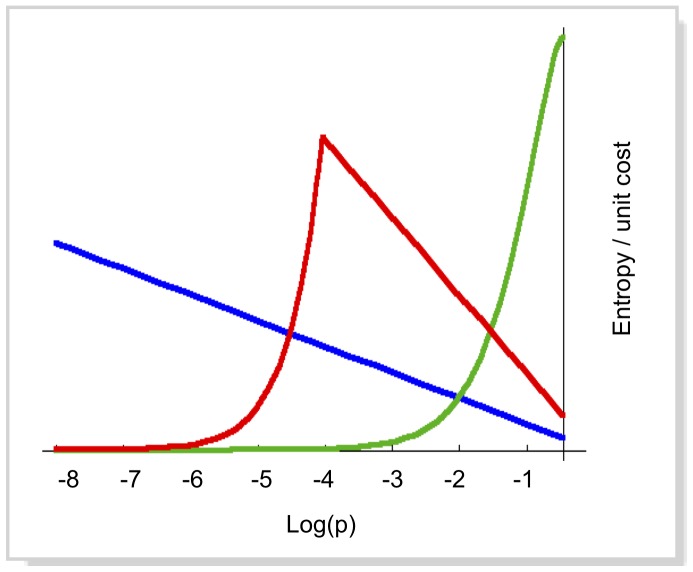
Entropy yield per unit cost, plotted as a function of the pattern probability (eq. 1). Blue curve: limited bandwidth and unlimited pattern storage capacity (W = 0.001, N = ∞); green curve: limited storage and unlimited bandwidth (N = 100, W = ∞); Red curve: limited bandwidth and storage (N = 100, W = 0.001)). Parameter values and the vertical scale are arbitrarily chosen for illustration.



As mentioned in the introduction, we assume that our system can recognize only a fixed number of pre-determined patterns. Let *p_i_* be the probability that a given portion of the input data matches a specific pattern *i*, out of a set *Q* of mutually exclusive patterns, such that ∑*p*_i_ *= *1, when *i* runs over all *Q*. The pattern-recognition system can be thought of as an array of *N* pattern-matching elements, each of them capable of recognizing the occurrence of the single pattern *i*, providing a single output bit, that signals the presence of the pattern in the input. This system would produce, on average, an information output equal to *−p_i_log(p_i_)* – that is, it is a source of entropy *−p_i_log(p_i_).* Neglecting correlations, the total entropy of the system is simply ∑*^N^_i_ −p_i_log(p_i_).* In absence of other constraints, maximization of the total entropy would be attained by simply including all possible patterns. This trivial solution implies transferring to the output the whole information in the original input, with just a change of format. The key to a meaningful answer is the explicit inclusion of the limitations of the system. The system can recognize up to a maximum number *N* of distinct patterns; to obtain the maximum entropy output under this constraint, patterns should be chosen to maximize the function *−p_i_log(p_i_)*, which peaks at *p = 1/e* ≈ 0.368. This is a large probability, and in practice it is likely to lead to selecting the patterns with the highest probability of occurrence in the input (see fig. 1). However, the output flux of the system is also bounded, due to bandwidth limitations, and the choice of the most probable patterns could quickly exceed this limit. In order to account for both constraints, we associate a “worst-case” cost to each pattern, defined as the larger of the “storage cost” 1/N and the “bandwidth cost” p_i_/W, where W is the maximum allowed total rate of pattern acceptance, ∑ *p_i_<W*. Therefore, an entropy yield per unit cost is given for each pattern by:

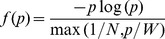
(1)The optimal performance of the filtering system is then attained by choosing the set of patterns such that *f(p_i_)>c*, where *c* is determined by the computational limitations: ∫_*f(p)>c*_ *δ(p)*d*p<N* and 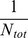
∫_*f(p)>c*_ *pδ(p)*d*p<W*, where *δ(p)* is the density of patterns having probability of occurrence *p*, normalized to the total number *N_tot_* of patterns in *Q*. The quantity 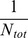
∫_*f(p)>c*_ *pδ(p)*d*p* is the average fraction of image elements that match successfully, and get preserved in the output - its inverse is the *compression factor* achieved by the filtering algorithm.

We have therefore an unambiguous and general recipe to determine the optimal set of patterns that a generic pattern-filtering system should use, in order to achieve maximum information preservation under the given constraints.

It is interesting to note that the function *f(p)* has a rather sharp maximum (a cusp, see fig. 1). As a consequence, the optimal set of patterns will be concentrated in a limited range of values of *p* around the maximum of *f(p)*, which occurs at *p = W/N*; it will therefore depend on both available storage size and bandwidth.

### Application to Real-time Data Acquisition in HEP

**Figure 2:**
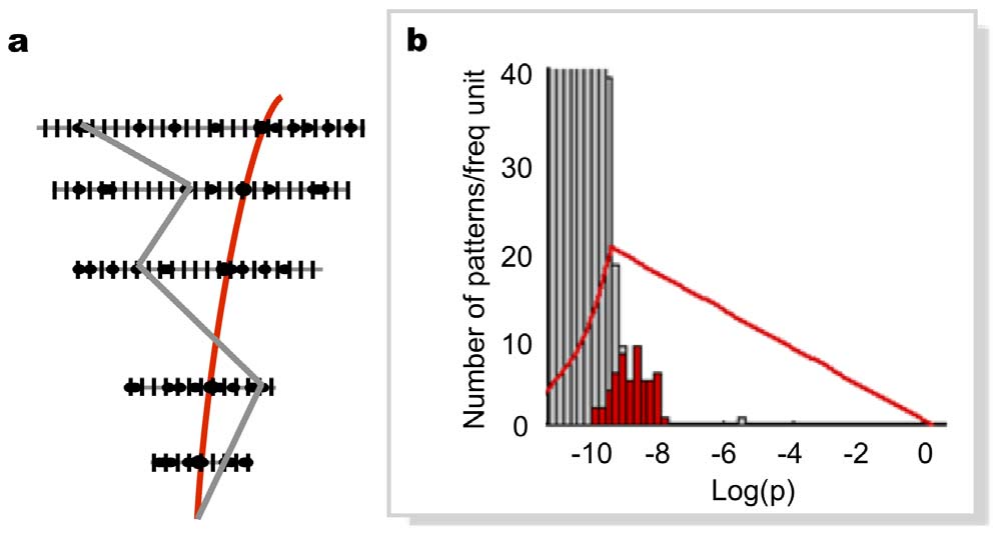
Monte Carlo simulation of track reconstruction and pattern filtering in a HEP particle detector. **a**, Schematic representation of a sector of a four-layers tracking detector, with simulated data (see Methods). Black dots represent measured positions where flying particles cross the detector layers - they can also be produced by random noise. Each layer is subdivided into a finite number of intervals (*bins*), delimited here by vertical bars. Every possible combination of bins (one on each layer) defines a *pattern* (grey line example). Only a small fraction of the patterns are compatible with the presence of a real particle (red line example), **b**, Probability distribution of the frequency of patterns (*δ(p)*) produced by a sample of simulated events of the type shown in (a) (grey histogram). The distribution of the sub-sample of patterns corresponding to valid particle trajectories is shown as a red histogram. The red curve is the function of eq. 1, with N = 50 and W = 0.15. The vertical red lines indicate the probability range selected by our model, using the constraint 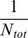 ∫_*f(p)>c*_ *pδ(p)*d*p<W*.

We can test the effects of the formula derived in the previous section in the above-mentioned case of data-reduction in HEP experiments with Associative Memory electronics. For simplicity we used a sample of simulated data with a Monte Carlo methods, rather then real data. This is a common practice in the field and will not affect our conclusions. We performed our tests with a detector structure and configuration that has been in actual use in particle physics experiments. For simplicity, we use the earliest and simplest configuration that has been in actual use, with four planar measuring layers [18]. Using the same computer code used in the real application, we produced simulated events, containing particles moving along circular trajectories, at a rate of about 50 per event. Only one sector of the 72 composing a whole detector was simulated. Realistic detector conditions were simulated by addition of random noise hits at an average rate of 1% per bin, and four “always on” hits due to defective detector channels. Measured coordinates on each layer of the detector are discretized in 500 µm bins. Every possible combination of bins (one on each layer) defines a “pattern” (fig. 2a). From the knowledge of the detector geometry and response characteristics, we computed a priori the set of all possible patterns corresponding to valid particle traversing the detector, as it is done in the real application in order to pre-program the pattern-finding device [18]. In a second step, we generated the probability distribution *δ(p)* of the frequency of all possible patterns, from a sample of 100,000 simulated events. We then proceeded to compare the probabilities of the valid patterns within the overall distribution with the prediction obtained from our own recipe outlined in the previous section (fig. 2b).

### Extraction of Optimal Visual Patterns from Natural Image Statistics

In applying our model to vision, we have considered the simplest possible set *Q* of base patterns, defined as all possible configurations of 3*3 square pixel matrices in black-and-white images (1-bit depth) (this discretization step is analogous to the pre-processing done to prepare data for Associative Memory devices). Although there are evidences of non-uniform distribution and characteristics of neurons within the same visual structure over the visual field carrying different information rates (see for instance [21], [22]), we assume a single pattern set, with a single spatial scale, to be valid for the whole image. This is clearly a simplification, but it should be adequate for our purpose of exploring the usefulness of the general principles of our model.

**Figure 3:**
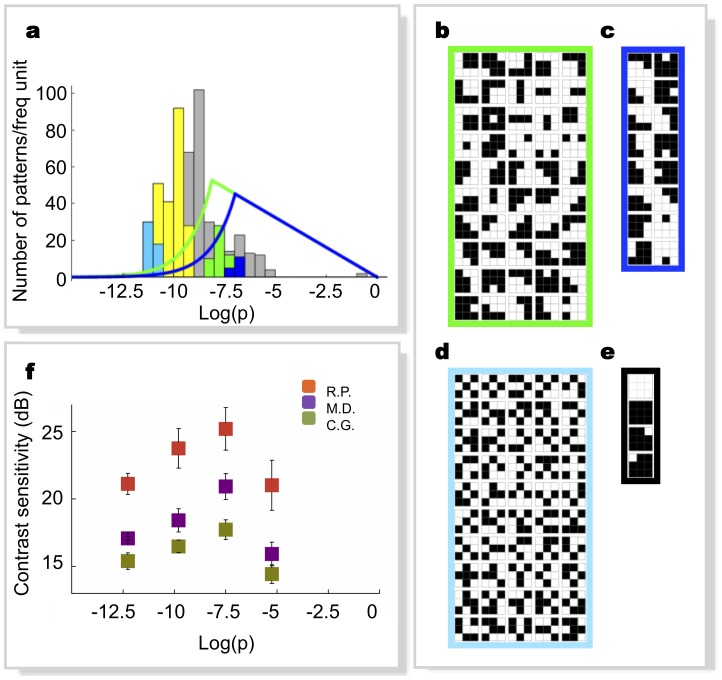
Human contrast sensitivity to visual patterns vs. model predictions. **a,** Probability distribution of the 512 possible 3×3 1-bit pixel patterns (grey histogram). The curves are the model selection functions (eq. 1) for W = 0.05 and two different values of N.(green: N = 50; blue: N = 15). Green and blue histograms are the probability distributions of corresponding selected patterns. Their actual bandwidth occupancies (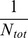
∫_*f(p)>c*_ *pδ(p)*d*p*) turn out to be slightly lower (respectively 0.025 and 0.015) than the imposed limit W. Cyan and yellow histograms are the distributions of low-probability patterns used in our measurements. **b,c,** Visualization of the pattern sets shown in (a), in green and blue respectively. **d,** Visualization of the lowest-probability patterns (discarded by our approach due to large storage occupation). **e,** Visualization of the highest-probability patterns (discarded due to large bandwidth occupation). **f,** Averaged sensitivity for detection of the patterns as a function of their probability, measured on three human subjects (different colors). Errors are determined by the fit (see Methods). The results of pairwise statistical comparisons (z tests, N = 100) amongst sensitivities plotted in (f) are: 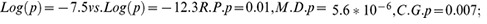 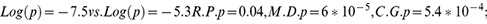 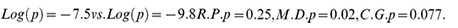

**Figure 4:**
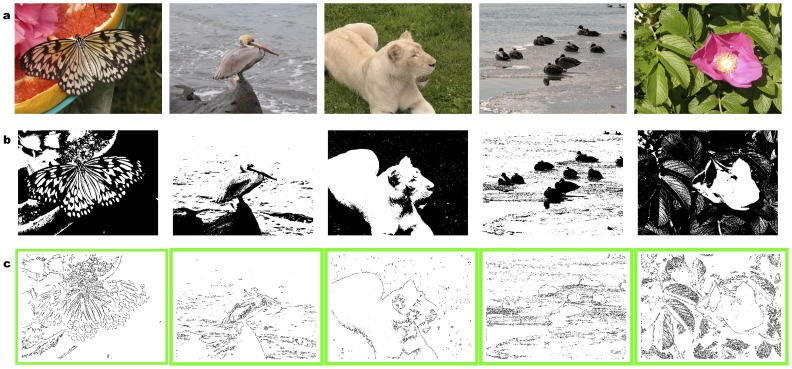
Examples of images from the database and sketches used. **a** Examples of full color natural images extracted from the database [23], available at: http://tabby.vision.mcgill.ca/html/browsedownload.html. **b** Digitized versions of images in (a). **c** Sketches obtained from the images in (b), by using the optimal pattern set of fig. 3b.

oWe then evaluated the probability distribution of the patterns in a set of natural images, and extracted the optimal set of patterns as per our recipe (fig. 3a). For this purpose we used a public database of 560 calibrated natural pictures [23] (see examples in fig. 4a). Each image (768×576 pixel) was digitized to 1-bit luminance (black/white), by setting the threshold at its median luminance value (fig. 4b).

The choice of the algorithm parameters (N,W) was based on the following considerations. Since the algorithm revolves on the idea of a strong compression at the minimum possible computational price, we decided to consider compressions of at least a factor 20, so we set W = 0.05 as a constraint, and we picked N = 16 as a “bare minimum” to be able to handle at least a few different spatial orientations. We take as a reasonable upper bound to N a value of 10% of all possible distinct patterns. Given that only 512 total distinct patterns are possible in our basic 3*3 model, we picked N = 50 as a limit.

All simulations and computations were implemented with Mathematica software (Wolfram research) on MacBook Pro computers.

### Psychophysical Methods

For all research involving human subjects, data were analyzed anonymously. All subjects were aware of the purposes of the study and gave written informed consent.

To measure contrast sensitivity (inverse of contrast thresholds), stimuli were presented to subjects with a 2IFC procedure. Each trial was composed by two intervals, both preceded by a tone, one containing the pattern on a mean luminance grey background, the other was set to mean luminance grey. Subjects (two naïve and one of the authors) were required to indicate the interval containing the pattern. For each subject and for each pattern, contrast thresholds (defined as the 75% probability of detecting a given pattern as a function of its Michelson contrast) were evaluated off-line with a 2 independent parameter MLE fit, cumulating data over 300 trials.

Contrast of patterns was randomly chosen from trial to trial within a set of predetermined values in the range 0.01 to 0.22 (“method of constant stimuli”). All patterns, 3.5 min/arc wide, were chosen randomly at each trial from the whole set and presented for 200 ms., in the center of the monitor (Barco Calibrator: frame rate = 80 Hz, resolution = 1280×962 pixel), via a 15 bit luminance resolution graphic board (VSG23F, Cambridge Research Systems). Their average luminance was normalized to the background (30 cd/m^2^). A smoothing algorithm (∇^2^(L) = 0, implemented with a relaxation method with 100 iterations) was applied to patterns to smoothly blend the outer pattern boundaries with the surrounding background. This is aimed at avoiding possible biases in the measurement due to the presence of spurious edges at the outer boundary of the pattern. Viewing distance was 60 cm.

For the discrimination experiment, we prepared “sketches” from natural images extracted from the same database used in determining the pattern set. These sketches were obtained by keeping in the thresholded image only those patterns corresponding to the chosen pattern set and blanking all other parts of the image. All possible 3×3 pixels patches, centered on every pixel of the image were considered (including overlaps).

In the discrimination experiment, sketches (23°×18° wide) were shown centrally to the subject using a Silicon graphics (frame rate = 100 Hz, resolution = 1152×864 pixel). In order to probe early stages of visual analysis [24], the presentation duration was 20 ms, and the stimulus was followed by a random noise mask (duration 750 ms, 23°×18° wide). Subsequently, two additional stimuli (19°×18° each) were presented side-by-side for 700 ms, one of them being the unfiltered image corresponding to the sketch, and the other a distractor, randomly selected from the dataset; both of them digitized to 1-bit. The subject was asked to identify the correct match with the sketch in a 2AFC procedure, by pressing a computer key.

In the control experiment, we used a full image with 256 grey levels instead of the sketch for the fast presentation, and the same image and a distractor in the second presentation, also with 256 grey levels.

Experiments were performed with three naive subjects and one of the authors, interleaving different conditions. Viewing distance for all experiments was 60 cm. (Luminances: white = 45 cd/m^2^, black = 1.4 cd/m^2^, medium gray = 13.8 cd/m^2^).

## Results

A significant difference between HEP applications and natural vision is that in HEP the reference set of patterns is completely defined by the a-priori knowledge of detector geometry and other details. We can compare what our model predicts to be the optimal pattern set with what has been actually implemented in HEP following completely independent methods. Comparison of these two sets shows that the patterns corresponding to real particles all fall within a limited range of intermediate probability within the overall distribution (fig. 2b). This implies that they could have been identified and selected according to eq. 1 (for an appropriate choice of parameters), that is, purely on the basis of statistical properties of the data. This remarkable fact is not exploited in current HEP applications, because one can usually rely on the a-priori knowledge of the meaningful patterns, although in principle it might find some applications (for instance, automatic adaptation to changes in detector positioning over time). In any case, the success of our model in identifying the right patterns in a HEP application encourages us to consider its application to the question of identification of salient features in natural vision, where the “right set of patterns” for the problem is not *a-priori* known.

Results for visual patterns extraction are shown in fig. 3 (a, b, c), for two different choices of N and W. Interestingly, it turns out that, independently of the precise choice of parameters, about 70% of the patterns selected by this algorithm can be classified as edges, bars, or end-stops, of various orientations (within the limitations of a 3*3 grid). Others are interpretable as corner detectors. Conversely, most of the patterns discarded by our selection have either an irregular structure resembling visual noise (fig. 3d), or uniform luminance, (fig. 3e), with lower resemblance to known visual features. In summary, biologically plausible features emerge here naturally from first principles, as the patterns that can be efficiently encoded by a system with finite computational resources.

While these results are suggestive, more direct evidence is needed that the human visual system actually assigns to these patterns a privileged role in its image-reconstruction process. To this purpose, we performed psychophysical measurements of contrast sensitivities for the detection of single isolated patterns. We tested a wide selection of all possible patterns, scanning the entire range of probabilities found in natural images (see Methods). Results (figure 3f) show that the contrast sensitivity of all subjects peaks within a limited probability range, in agreement with the predictions of our model.

It is worth noting that these results are not simply a consequence of the band-pass behaviour of the human contrast sensitivity as a function of spatial frequency. Although there is a mild correlation between the probability of pattern occurrence and their spatial frequency content (the rarer patterns containing on average more of the higher spatial frequencies), there is a significant overlap of spatial frequency content between the patterns over the whole range of fig. 3f. More importantly, the spatial frequency spectrum of all our patterns lies entirely above the frequency of maximum human sensitivity. Our range of spatial frequencies is between 9 cycles/deg and 27 cycles/deg, while the maximum sensitivity lies at about 7 cycles/deg in our illumination conditions [25]. Therefore, spatial frequency sensitivity considerations would predict a low-pass behaviour, resulting in an increasing function in fig. 3f, which is very different from what we observe.

**Figure 5:**
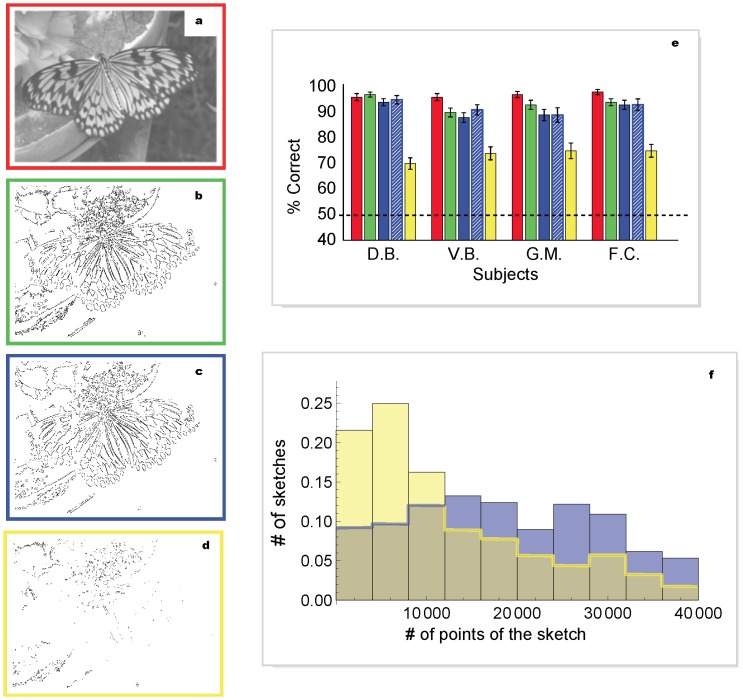
Examples of sketches obtained from different pattern sets. **a,** Original 256 grey-levels image. **b**, Sketch obtained from the optimal pattern set of fig. 3b. The corresponding compression factor is 40, and its information content is 9.8% of the original. **c,** Sketch obtained from the optimal pattern set of fig 3c. The corresponding compression factor is 67 and its information content is 5.5% of the original. **d,** Sketch obtained from the 244 low-probability pattern set (fig. 3d shows a sub-sample); information (5.5%) and compression (factor 90) are similar to (c). **e,** Percentage of correct discrimination for sketches obtained as in (b), (c), (d) (green, blue, yellow bars respectively) and 256 grey-levels images as controls (red bars), for four subjects. The striped blue bar represents results obtained from the same dataset shown in blue, after reweighting the data to match the distribution of the number of patterns of the yellow dataset. **f,** Distributions of the number of points found in the sketches for the two sets in (c) and (d), shown with the same color code. The distributions are taken over the entire image database of our study. Each data point represents 300 trials. The black dashed line indicates chance performance. Error bars are s.d. The results of pairwise statistical comparisons (binomial tests) amongst performances plotted in (f) are: red vs. green: D.B. p = 0.96, V.B. p = 0.06, G.M. p = 0.16, F.C. p = 0.13; red vs. blue: D.B. p = 0.48, V.B. p = 0.009, G.M. p = 0.003, F.C. p = 0.08; blue vs. green: D.B. p = 0.3, V.B. p = 0.7,G.M. p = 0.3, F.C. p = 0.9; blue vs. yellow: D.B. p = 9.8*10^−12^, V.B. p = 2*10^−5^, G.M. p = 0.002, F.C. p = 1.1*10^−7^.

The model, however, makes a stronger statement than an enhanced sensitivity: it predicts that early visual processing exclusively utilizes those parts of the images matching predicted reference patterns to create a compressed internal representation for fast processing. To test this prediction, we created “sketches” from our images, by keeping only those patches of the binarized image matching one of the patterns of a given reference set, dropping all other parts. These sketches represent our prediction for the output of the early visual processing stage we are trying to model. Inspection of the examples shown in fig. 4 and 5 show that, independently of the specific choice of parameters, the sketches obtained from optimal pattern sets appear to retain most of the salient features of originals, in spite of a substantial reduction of information. To quantify this qualitative observation, we measured the effectiveness of these sketches in allowing human observers to identify natural images under fast viewing conditions (20 ms) (see Methods). If the early visual system really selects only those specific patterns for its processing, than our sketches should elicit nearly the same response as complete images.

All subjects were able to identify the original images from which these sketches were extracted with extremely high accuracy (fig. 5e). Even more important, performance was comparable to measurements obtained in a control experiment using the fully detailed original images in place of their sketches. Subjects reported that they could not tell whether originals or sketches had been shown to them in these fast presentations. In contrast, we found that sketches built based on alternative, non-optimal pattern sets yield a much worse representation of the features perceived as salient by human observers, even when constructed to contain the same amount of information of our “optimal” sketches (fig. 5d vs. 5c). The ability of subjects to identify original images from these alternative sketches is much worse than with our optimal sketches.

**Figure 6:**
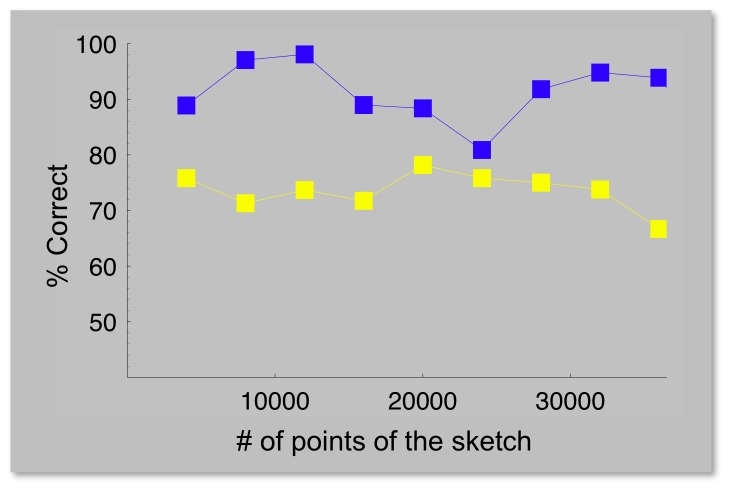
Percentage of correct discrimination, averaged over all subjects, plotted as a function of the number of matched patterns, for the same data as in fig. 5c and 5d.

It must however be noted that the distributions of the number of points found in these two sets, taken over the whole image database (fig. 5f), have different average values: ∼14000 for the alternative set and ∼24000 for the set predicted by the model. Therefore, we performed an additional test to exclude that the observed difference in average performance might be due to the difference in the average number of visible points. For each experimental trial, we reweighted in the final average the data taken with the pattern set predicted by the model by a factor equal to the ratio of the probability distributions of the two sets, i.e. the ratio of the heights of the histogram bars in (fig 5f) corresponding to the number of points of the sketch presented. In this way, the density distribution of the predicted patterns is forced to match that of the rarer patterns, and any possible dependence of the result on the density of the image gets equalized between the two sets. The results (fig. 5e) show that the reweighting procedure has no significant effect (it shifts the results by less than one standard deviation). To further investigate the issue, we replotted our data differently, splitting the trials in different sets, according to classes defined by the number of points in the sketches (fig. 6). The difference in discrimination performance between the two sets is apparent over the whole range: even densely-populated sketches made of rare patterns are less visible than those from the standard set confirming that the number of displayed points plays no measurable role in our measurements.

All these results support the central idea of our model, that the features identified in natural images by our mathematical model carry most, or all of the information that the visual system is capable of using under fast viewing conditions.

## Discussion

In vision research literature, it is usually taken for granted that edge-detection is amongst the main objectives of visual processing. Many models have been proposed, aimed at attaining the best performance in detecting objects contours using biologically plausible elements, based on a-posteriori knowledge of physiological details [14]-[16]. In this work we found that the edge-detection functionality in itself, follow directly from very general principles, as the optimal solution for fast processing, when dealing with an information bottleneck and limited computational resources.

It is interesting to compare our results to the work of Olshausen and Field [13], who succeeded in deriving biologically plausible basis functions for visual representation, based on considerations of information efficiency. In spite of some superficial similarities, there are important differences. Their work aims explicitly at reproducing the original luminance map as closely as possible, based on a minimum chi-square criterion, and utilizes a number of free parameters to achieve the best results: it can be described as aiming to an “almost-lossless” compression, akin to image compression algorithms.

In the present work, we consider instead a scenario where a strong reduction of information is needed, and look at entropy maximization without regard to fidelity of reproduction – there is no choice of free parameters, and the solution is uniquely determined by the system limitations, resulting in selection of a restricted number of salient features. Our figure of merit for the results is recognisability by human observer, not the accuracy of the raw luminance map (which is definitely much worse than in Olshausen and Field [13]).

In comparing to past studies specifically devoted to the extraction of features, we note that several of them argued that the visual system evolved to detect the features of natural images, and devotes resources to this detection in proportion to the probability of feature occurrence [26], [27]. Results here, on the other hand, show that principles of computational efficiency lead to a somewhat different algorithm: resources are devoted to features with an *intermediate* probability of occurrence. Discarding the most probable input configurations is necessary to fit within the bandwidth limitations of the next processing stage. As an example, the most common visual patterns, uniform luminance patches, which are obviously inefficient to encode, are automatically rejected by our model.

A unique feature of our model is that there exists at least one concrete example of a detailed successful implementation (in electronic devices) so there is no question that it is actually implementable. It is also not hard to conceive that it can be implemented in a neural network: it is well known that recognizing the presence of a certain set of discrete input patterns is within the typical capability of a neural network [28].

One may wonder whether this process of “data compression by pattern selection” happens within the visual system. In principle it does not need to be localized to any specific area, as it might be distributed along a path, but several evidences indicate as most likely candidate the primary visual cortex. Only a modest amount of compression occur in transporting information from the retina to V1, that is actually the most extended visual area, and has a large neural ratio with respect to the retina. This has a direct analog in the Associative Memory, that is used in performing pattern selection in HEP applications, which is the most conspicuous part of the system and uses the largest fraction of electrical power. These characteristics are not accidental, but a necessary consequence of a pattern-selection organization. It must also be noted that many of the patterns selected by our algorithm are good approximations, within the limitations of a simple 3*3 grid, of the structure of some types of receptive fields of neurons in primary visual areas [29] [30], so their involvement in their recognition and selection appears plausible. All of this is consistent with the idea, previously advanced, that the function of V1 is to create a “bottom-up saliency map” enabling a “lossy pre-attentive selection of information”, so that data rate can be further reduced for detailed processing [31], [32]. This is also consistent with the involvement of V1 in fast natural object recognition [2], and its activation in fMRI experiments of fast image viewing in conditions similar to ours [33].

Another important question is how the visual system could have developed to use the optimal pattern set. One attractive aspect of our model is that it allows for easy algorithms for unsupervised learning. The optimal patterns have probabilities falling within a limited range. A system that is initially sensitive to a wide variety of patterns could converge towards optimality simply by discarding patterns that occur too rarely, or too frequently, in the input. This process might even happen during normal activity, allowing for continuous updating and adaptation to changing external conditions**.** It is interesting to note while initial neural network learning models using a Hebbian rule would lead to unlimited reinforcement of patterns presented with larger frequencies, most modern learning models include an anti-Hebbian component (e.g. BCM [34]) to ensure stability, and this leads to maximize sensitivity to an intermediate range of frequencies, consistent with the view presented here.

Several evidences exist of a continuous adaptation of the visual system to external stimuli. Exposure to visual patterns is necessary for improvement of visual function in infants [35] and for normal development of visual cortex in kittens [36], [37], as it ensures that the feature-detecting properties of cortical cells are matched to the statistical properties of features in the visual environment [37]–[39]. Even in adulthood, repeated exposure or training to visual patterns has been shown either to sharpen perceptual abilities for novel stimuli [40], [41], or to allow adaptation to familiar stimuli [42], [43]. This plasticity might have significant evolutionary benefits, for example enabling humans to sharpen their ability to discriminate facial features of their own race [44].

All such phenomena are consistent with the behavior of a system striving to achieve and maintain the optimal performance compatible with its limited computational power, by continuous retuning of its reference patterns to an intermediate probability range. This same idea might also provide an explanation for the observed ability of adaptation to very non-natural visual environments (medical images [45] or other specialized fields [46]), which would be otherwise difficult to explain or justify on its own merits.

### Conclusions

We have seen that a simple abstract model of information-optimal pattern filtering is capable of producing detailed predictions, that match very well both the behavior of the visual system under fast viewing conditions, and the behavior of artificial data processing devices that have been designed and developed following entirely different principles. What the two systems have in common is a need for large data reduction under the constraint of limited computational resources. It appears that the constraints on these systems are so severe that they do much more than simply limit their performance: they seem to take the dominant role in shaping what the system selects (“perceives”) as *relevant features* in the input, so that they allow us to predict them from simple information-theoretic considerations. One could argue that the idea of a pattern-filtering architecture has attained success in HEP applications due to the pressure of the need for high-speed large-scale data reduction at reasonable costs [20], and that the optimization of the use of neural computational resources must have played an important role in natural selection [47], leading to an example of convergent evolution of natural and artificial systems towards the same, optimal solution. As we discussed, this solution can be reached via an unsupervised learning process, and can be self-adapting to changing external conditions, which facilitates its implementation in natural systems. It would be interesting to investigate experimentally whether such learning process actually exists, that we have just argued to be plausible. These ideas are quite general, and it would also be interesting to study other perception systems with this approach to see if their features can be understood from the same principles, and to what extent the ecological limitations to their processing power determine what the system categorizes as “meaningful”.
